# Mutations in SilS and CusS/OmpC represent different routes to achieve high level silver ion tolerance in *Klebsiella pneumoniae*

**DOI:** 10.1186/s12866-022-02532-y

**Published:** 2022-04-25

**Authors:** Charlotte A. Woolley, J. Mark Sutton, Matthew E. Wand

**Affiliations:** Technology Development Group, UKHSA, Research and Evaluation, Porton Down, Salisbury, Wiltshire, SP4 0JG UK

**Keywords:** *Klebsiella pneumoniae*, Silver tolerance, SilS, CusS, OmpC, Silver nitrate

## Abstract

**Background:**

Silver ions have potent broad-spectrum antimicrobial activity and are widely incorporated into a variety of products to limit bacterial growth. In Enterobacteriaceae, decreased silver susceptibility has been mapped to two homologous operons; the chromosomally located *cus* operon and the plasmid based *sil* operon. Here we characterised the mechanisms and clinical impact of induced silver tolerance in *Klebsiella pneumoniae*.

**Results:**

In *K. pneumoniae* carriage of the *sil* operon alone does not give elevated silver tolerance. However, when exposed to increasing concentrations of silver nitrate (AgNO_3_), *K. pneumoniae* strains which contain the *sil* operon, will preferentially mutate SilS, resulting in overexpression of the genes encoding the RND efflux pump *silCBA*. Those strains which do not carry the *sil* operon also adapt upon exposure to increasing silver concentrations through mutations in another two-component regulator CusS. Secondary mutations leading to disruption of the outer membrane porin OmpC were also detected. Both routes result in a high level of silver tolerance with MIC’s of >512 mg/L. When exposed to a high concentration of AgNO_3_ (400 mg/L), only strains that contained the *sil* operon were able to survive, again through mutations in SilS. The AgNO_3_ adapted strains were also resistant to killing by challenge with several clinical and commercial silver containing dressings.

**Conclusions:**

This study shows that *K. pneumoniae* has two possible pathways for development of increased silver tolerance but that the *sil* operon is preferentially mutated. This operon is essential when *K. pneumoniae* is exposed to high concentrations of silver. The potential clinical impact on wound management is shown by the increased survivability of these adapted strains when exposed to several silver impregnated dressings. This would make infections with these strains more difficult to treat and further limits our therapeutic options.

**Supplementary Information:**

The online version contains supplementary material available at 10.1186/s12866-022-02532-y.

## Introduction

With the exponential rise of antimicrobial resistance, alternative treatments like heavy metals are experiencing an increased interest in their potential to treat infections [1]. Silver in various forms has been incorporated into materials for clinical and non-clinical use, ranging from wound dressings and surgical implant coatings to personal care products [2]. However, this rise in the use of silver has raised concerns over the emergence of microbial resistance against silver and other heavy metals [3] as well as the potential for cross-resistance to conventional antibiotics.

Silver is not required for bacterial metabolism, therefore silver ions (Ag^+^) have potent broad-spectrum bactericidal activity [4, 5]. Silver ions will target key functional groups in bacterial enzymes and proteins e.g. thiol groups. They also cause destabilisation of the bacterial cell membrane through release of K^+^ ions and incorporation of silver into the membrane structure [6]. Other modes of antimicrobial action include damage of intracellular structures such as DNA and ribosomes, and oxidative stress caused by generation of reactive oxygen species (ROS) and free radicals [7, 8].

Increased tolerance to silver has been shown for several Gram-negative organisms [9–12] and this has been mapped to changes in two operons. The first is the chromosomally encoded *cus* operon which consists of a heavy metal RND efflux pump coupled with a metallochaperone (CusCFBA) regulated by a divergently expressed two-component regulator (CusRS) [13]. Increased silver tolerance attributed to the *cus* operon is also linked to decreased membrane permeability [14]. *Cus*-mediated active efflux of silver ions was synergistically enhanced following the loss of one or both of the major porins OmpC and OmpF [12, 15].

The second is a plasmid based encoded *sil* operon whose function is predicted based on homology to the *cus* operon [16]. The *sil* RND efflux pump component (SilCFBA) shares a >80% homology to CusCFBA and is regulated by a two-component regulator SilRS [12, 17]. Three additional ORFs with no *cus* homologue are also found in the *sil* operon: SilP, a putative P-type ATPase transporter, and SilE and SilG, periplasmic sequestration proteins and metallochaperones [12, 18, 19]. Mutations within the regulators *silR* and *silS* have been shown to increase expression of the *sil* operon coupled with a decrease in silver susceptibility [12].

*Klebsiella pneumoniae* is an opportunistic pathogen belonging to the Enterobacteriaceae family. The species is prominent in causing wound infections as well as hospital and community-acquired urinary and respiratory tract infections [20]. Moreover, *K. pneumoniae* has an antimicrobial resistance profile with multi- and extensively drug-resistant strains (MDR and XDR) regularly isolated from patients [21].

In this study, we show that in *K. pneumoniae* the *sil* operon is essential for survival following direct exposure to high levels of silver ions and that the primary mechanism of adaptation to increased concentrations of silver is through mutations in the sensor kinase SilS leading to increased expression of the *silCFBA* operon. For strains that do not contain a *sil* operon, survival at low silver concentrations was through alterations in another sensor kinase CusS. A secondary mutation in the outer membrane porin OmpC was essential in these strains for survival at high silver concentrations. The potential clinical impact of this decreased silver susceptibility in treatment of infections was addressed using a range of prescribed and commercially available silver-containing dressings and this showed that the silver-adapted strains are resistant to killing by all dressings.

## Results

### The absence of the *sil* operon does not correlate with increased susceptibility to silver

To evaluate whether strains of *K. pneumoniae* that possessed the *sil* operon were more intrinsically resistant to silver, the MIC/MBC levels to silver nitrate (AgNO_3_) and silver sulfadiazine (AgSD) from a range of strains were assessed. These included strains from clinically important sequence types ST14/15, ST23, ST101 and ST258 (Supplementary Table S1). The MIC values for those strains containing a *sil* operon (40/61) ranged from 8- >512 mg/L with a mode of 16 mg/L for AgNO_3_ and a range of 32- >512 mg/L with a mode of 32 mg/L for AgSD. For those strains lacking a *sil* operon (21/61) the MIC range was 8–64 mg/L with a mode of 32 mg/L for AgNO_3_ and a range of 32–256 mg/L with a mode of 128 mg/L for AgSD. When the genome sequences of the two strains (MGH 78578 and CFI_123_NDM1OXA232) which had high MIC values (>512 mg/L) were interrogated they were found to have unique mutations in SilS.

### The *sil* operon protects against challenge with a high dose of silver

**Fig. 1:**
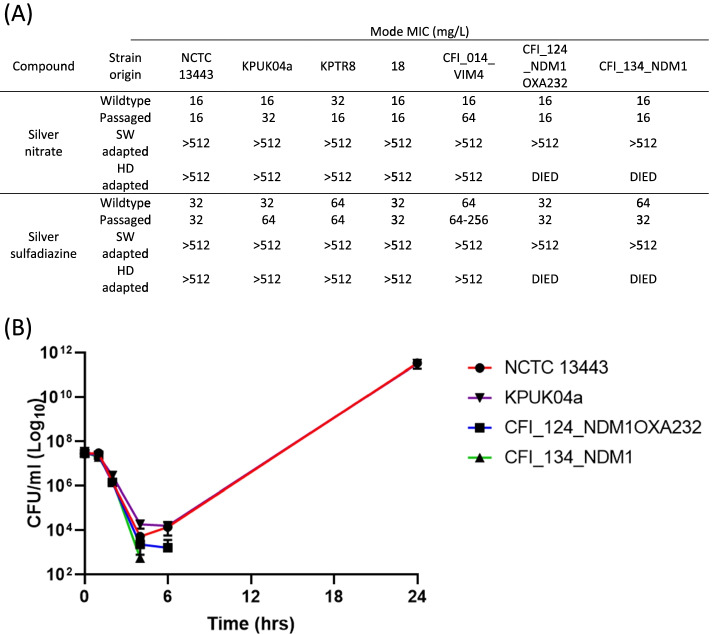
*K. pneumoniae* strains containing the *sil* operon can adapt to the presence of AgNO_3_. **A** MIC values for AgNO_3_ and AgSD of pre-exposure (Wildtype), repeatedly passaged in blank media without AgNO_3_ (Passaged), passaged in increasing concentrations of AgNO_3_ (SW adapted), and challenged with a single high dose (400 mg/L) of AgNO_3_ (HD adapted) strains. **B** Time kill showing generation of increased silver tolerance after 24 h exposure to above MIC concentration of AgNO_3_ (64 mg/L) for two strains (NCTC 13443 and KPUK04a) which contained a plasmid with the *sil* operon. Those strains (CFI_124_NDM1OXA232 and CFI_134_NDM1) which did not possess a *sil* operon containing plasmid died after 6 h exposure

To assess the importance of *sil* operon carriage in adaption to silver, seven genetically similar *K. pneumoniae* strains from the clonal complex 15 (CC15) consisting of a mixture of ST14/15 isolates, and including isolates which lacked the *sil* operon, were exposed to increasing stepwise (SW) concentrations of AgNO_3_. All strains were able to adapt and survive in the highest concentration of AgNO_3_ tested; subsequent MIC analysis showed a significant increase to >512 mg/L for both AgNO_3_ and AgSD (Fig. 1A).

The same seven isolates were also challenged with a supra-MIC (HD) concentration of AgNO_3_. Only five out of the seven isolates survived; the two isolates which died (CFI_124_NDM1OXA232 and CFI_134_NDM1) were the only isolates which lacked the *sil* operon. Again, those isolates which survived showed a substantial MIC increase to >512 mg/L for both AgNO_3_ and AgSD (Fig. 1A). Time kill analysis showed that AgNO_3_ is initially highly bactericidal with a greater than 3-log reduction in viable bacteria for all strains tested. However, the large increase in viable bacteria for those strains which possess the *sil* genes between 6 and 24 h indicates the generation of isolates with increased silver tolerance (Fig. 1B).

### Increased silver tolerance is linked to mutations in the two component sensor kinases SilS and CusS which leads to overexpression of the *silCFBA* or* cusCFBA* operons respectively

Subsequent genome analysis of strains which had developed increased silver tolerance showed that a range of mutations were observed in *silS* (Table 1) for both SW and HD adaptations. For those strains without the *sil* operon SNPs were found in *cusS* and *ompC*.

**Table 1:** Genetic mutations found in *K. pneumoniae* isolates generated from stepwise (SW) and single high dose (HD) exposure to AgNO_3_

Strain	Adaptation	SilS^a^	CusS	OmpC	Other
NCTC 13443	SW	A13V, W353R			
	HD	L12F			E46V molecular chaperone
KPUK04a	SW	L322Q			
	HD	G210E			W57R malate dehydrogenase
KPTR8	SW	A352D			
	HD	F321C			
18	SW	S304Y			
	HD	A352D, K255N			E650H TonB-dependent siderophore receptor
CFI_014_VIM-4	SW	Deletion 6 nucleotides after n1309			
	HD	S196I			P49T ABC transporter substrate binding protein
CFI_124_NDM-1 OXA-232	SW		L9Q	transposon insertion in *ompC*	
	HD (Died)				
CFI_134_NDM-1	SW		P273L	16 kb fragment deletion including *ompC*	
	HD (Died)				

^a^Where two mutations are shown it is because they are in different sequenced clones

Real time PCR analysis on selected strains from the SW adaptations showed that those strains which contained mutations in *silS* (13443 Ag and KPUK04a Ag) had greatly increased expression of *silA* (over 250-fold) and a smaller fold-increase for *silS* (18 fold). For *cusA*, one strain (13443 Ag) showed a large increase in expression (41.6-fold) but in the other strain (KPUK04a Ag) no increase was observed. Strains which contained mutations in *cusS* and *ompC* (CFI_124_NDM1OXA232 and CFI_134_NDM1) predictably correlated with a substantial increase in *cusA* expression (over 250-fold) and a decrease in *ompC* expression (over 30-fold) (Supplementary Table S2).

**Fig. 2:**
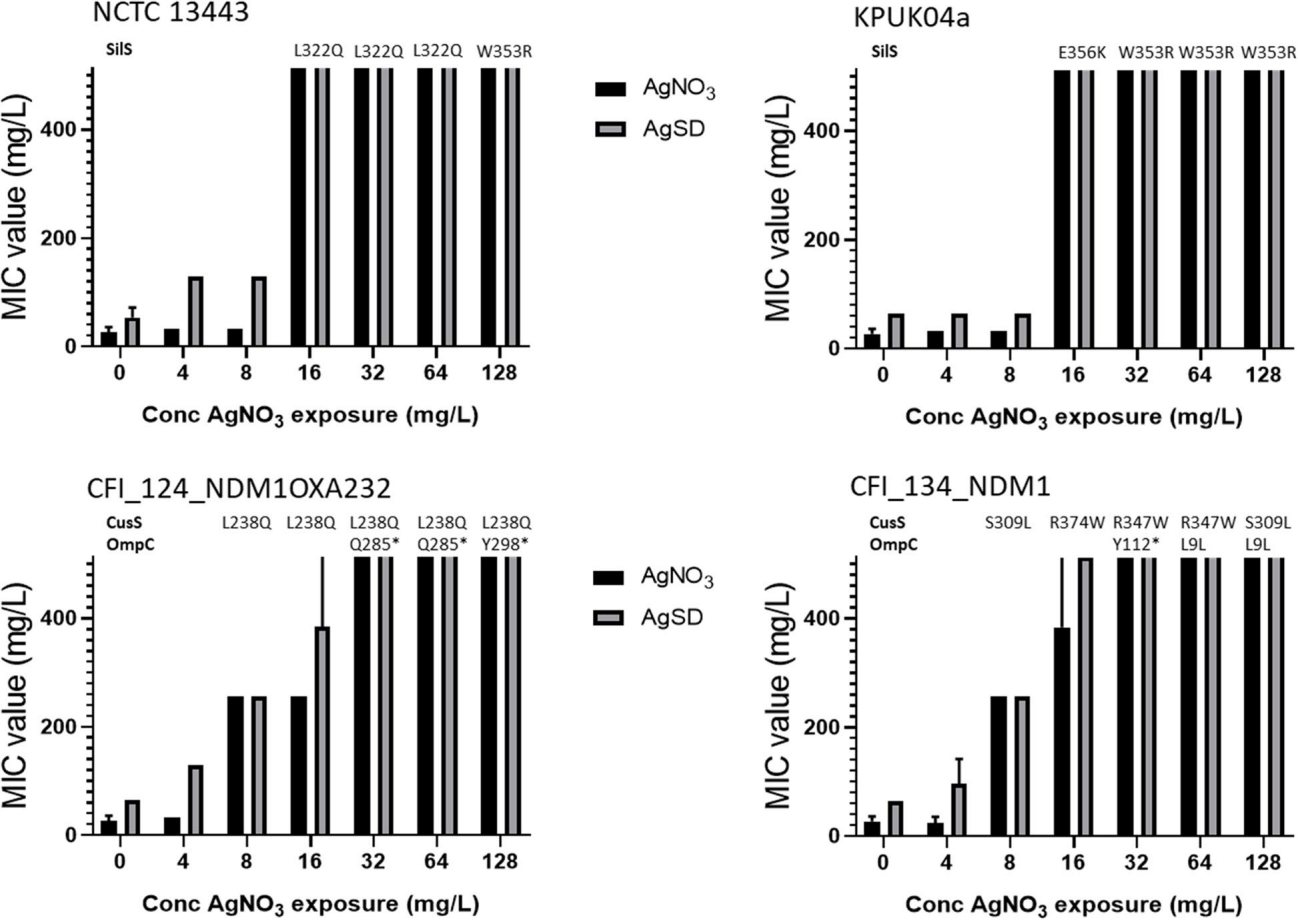
In strains which don’t contain the *sil* operon mutations in CusS are observed at sub-MIC AgNO3 exposure concentrations whereas mutations in OmpC were only found after exposure to supra-MIC silver concentrations. Strains which contain a *sil* operon (NCTC 13443 and KPUK04a) and strains which don’t (CFI_124_NDM1OXA232 and CFI_134_NDM1) were adapted to the presence of silver using a stepwise adaption method as already described where the AgNO_3_ concentration was doubled every two days starting at 4 mg/L and ending at 128 mg/L. At each exposure concentration the MIC values to AgNO_3_ (black bar) and AgSD (grey bar) were measured and compared to pre-exposure values (0 mg/L). Cultures from individual exposure concentrations were streaked onto TSA plates and individual colonies whole genome sequenced. Identified CusS, SilS and OmpC mutations are shown above the AgNO_3_ challenge concentration where they were observed. Values plotted are the mean of three repeats and the error bars represent the standard deviation (highest concentration tested 512 mg/L)

To understand if the isolation of mutations in SilS and CusS/OmpC correlate with a change in the MIC value to silver compounds, four strains were again stepwise adapted to AgNO_3_. At each concentration exposure, the population MIC to AgNO_3_ and AgSD was measured and isolates were taken and whole genome sequenced. For the two *sil* operon containing strains (NCTC 13443 and KPUK04a) mutations in SilS were detected at exposure to AgNO_3_ concentrations at the respective wildtype MIC level and above (16 mg/L and upwards). When these SilS containing mutants were tested, the subsequent MIC values were >512 mg/L for both AgNO_3_ and AgSD (Fig. 2). For those strains without a *sil* operon (CFI_124_NDM1OXA232 and CFI_134_NDM1) mutations in CusS were observed at exposure levels below the MIC value (16 mg/L) and gave rise to a slight increase in tolerance to AgNO_3_ (fourfold increase). OmpC mutations were only found at higher AgNO_3_ challenge concentrations (above MIC; 32 mg/L and upwards). Strains containing mutations in CusS and OmpC showed MIC levels for AgNO_3_ and AgSD of >512 mg/L (Fig. 2). Usually the OmpC mutations detected were the introduction of premature stop codons, although there was one synonymous mutation identified (L9L – CTG to TTG). Analysis of the *K. pneumoniae* codon usage showed that CTG is substantially the most common codon with a frequency of 70.9/thousand in *K. pneumoniae* MGH 78578; TTG has a frequency of 6.8/thousand. Presumably this synonymous mutation affects the expression of *ompC* with the introduction of a rarer codon close to the start of the gene, although this wasn’t tested.

**Fig. 3:**
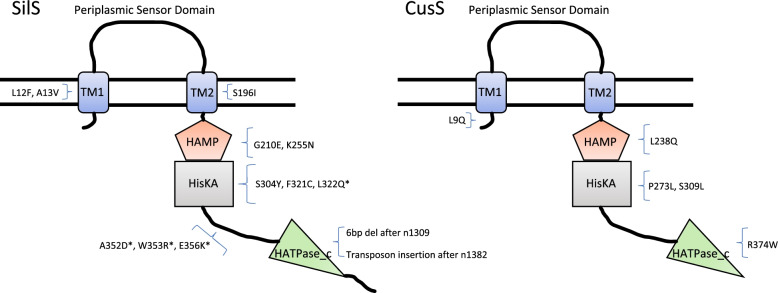
Schematic diagram of sensor histidine kinase SilS and CusS showing location of all mutations found in *K. pneumoniae* strains generated from adaptation experiments, in relation to domain structure. TM1, Transmembrane region 1 (12–34 SilS; 15–34 CusS); TM2, Transmembrane region 2 (188–207 SilS; 184–206 CusS); HAMP, putative regulator of phosphorylation (208–261 SilS; 204–257 CusS)); HisKA, dimerization/histidine phosphotransfer (262–328 SilS; 258–324 CusS); HATPase_c, ATP binding domain (372–483 SilS; 368–477 CusS). * indicates that this mutation was observed in more than one strain

Comparison of SilRS and CusRS sequences from all ST14/15 strains showed that was a high degree of homology between strains, especially for CusRS (Supplementary Table S3). Variation within SilSR, particularly for strains CFI_123_NDM1OXA232 and CFI_145_NDM1 may indicate acquisition of different plasmids. SilS sequence strain variations occurred predominately within the periplasmic sensor-domain. However, no mutations were observed in this region in strains adapted to the presence of silver, suggesting that variations within this domain may not significantly impact function. Mutations in SilS and CusS showed that, particularly for SilS, whilst they were not confined to one domain or region there were areas where mutations were more commonly found (Fig. 3) e.g. mutations were observed in multiple strains around amino acids 352 to 356 which is part of the cytoplasmic linker region. The highly silver tolerant strain CFI_123_OXA232NDM1 which was part of the initial screen also had a variation in SilS in this region (E355K).

### Silver adapted strains are not affected by efflux pump inhibitors

Since the *sil* and *cus* operons both contain an RnD efflux pump, we wanted to understand whether the presence of the EPI inhibitors carbonyl cyanide 3-chlorophenylhydrazone (CCCP) and phenylalanine-arginine β-naphthylamide (PaβN) affected silver tolerance in *K. pneumoniae*. Results showed that whilst the addition of PaβN had no effect on the MIC levels, CCCP was able to reduce the MIC to AgNO_3_ and AgSD for all parental strains by at least eightfold (Supplementary Table S4). For the adapted strains, only two, KPTR8 Ag and CFI_014_VIM4 Ag, showed any reduction in MIC with the addition of CCCP. It is not clear why these specific mutations retain susceptibility to CCCP but in both cases the MIC of the AgNO3 adapted strains after CCCP addition remained above that of the respective parental strain. The addition of the membrane permeabilizer Polymyxin B nonapeptide (PMBN) also did not alter the MIC for AgNO_3_ and AgSD for either adapted or non-adapted strains.

### Phenotypic characterisation of silver tolerant mutants

To comprehend whether acquisition of increased silver tolerance led to changes in biological fitness the growth of all strains from the SW adaptation was assessed. There was no significant change in the growth of adapted strains versus non-adapted strains (Supplementary Figure S1). Acquisition of increased silver tolerance may lead to either increased susceptibility or cross resistance to other antimicrobial compounds. A selection of antimicrobial agents including frontline antibiotics for treatment of *K. pneumoniae* infections as well as several cationic disinfectants and antiseptics used for infection prevention were tested. Since a common feature of ST14/15 K*. pneumoniae* isolates is the MDR phenotype, it was difficult to observe potential changes in acquisition of decreased susceptibility to antibiotics. Antimicrobials where efflux mediated resistance is problematic were prioritised since the *sil* and *cus* systems encode an efflux pump. There were only sporadic instances of changes (greater than twofold) in antimicrobial susceptibility with KPTR8 Ag showing increased susceptibility to doxycycline, chlorhexidine and DDAB. (Supplementary Table S5).

### Clinical impact of silver tolerant strains

**Fig. 4:**
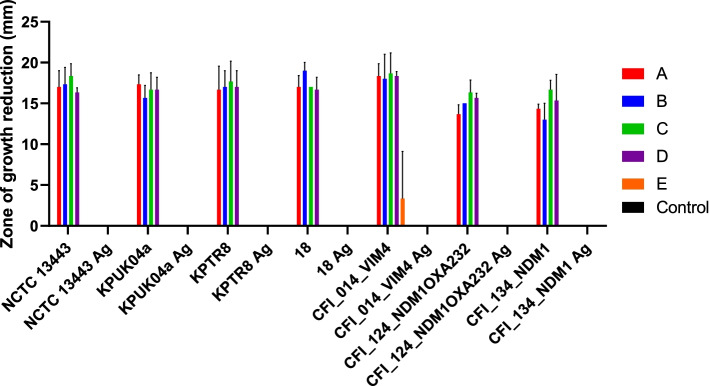
Silver dressings are ineffective against *K. pneumoniae* silver adapted strains. The susceptibility of seven wildtype and their stepwise-adapted counterparts to four clinically (**A**, **B**, **C**, **D**) and one commercially (**E**) available silver dressing were tested using zones of clearance. Values plotted represent the mean diameter of growth reduction from three independent experiments. Error bars represent standard deviation. Ag: Adapted strain

To understand the clinical impact of adaptation to silver by *K. pneumoniae* the ability of several clinical and commercially available silver dressings to reduce the growth of the non-adapted and adapted ST14/15 strains was measured. All clinical dressings produced a zone of growth reduction for all non-adapted strains, but the commercially-available dressing had no effect. For the adapted strains, regardless of whether the adaptation to silver was through mutations in SilS or CusS/OmpC, no growth reduction was observed after the application of any of the dressings (Fig. 4).

## Discussion

In this study we have characterised the acquisition of increased silver tolerance in *K. pneumoniae* and show that this is due to mutations in SilS or, for those strains without the *sil* operon, through mutations in CusS/OmpC. Mutations in these regulators led to subsequent overexpression of their respective divergently expressed operons, e.g. those strains that contained mutations in SilS were found to have highly upregulated *silCFBA* as shown by *silA* expression levels. Strains with mutations in CusS and OmpC had increased *cusCFBA* and decreased *ompC* expression levels. The *sil* operon was essential for the survival of strains exposed to supra-inhibitory concentrations of AgNO_3_. Mutations in either SilS and CusS giving rise to high level silver tolerance have been shown before in adaptation to silver [9, 12] and indeed some of those aforementioned mutations are replicated here, confirming their importance. Comparison with other two component sensors, e.g. EnvZ enables us to predict some of the likely functions of the SilS and CusS mutations. Those mutations found in the transmembrane domains are likely to be involved in uncoupling signalling from silver sensing in the periplasmic sensor domain [22]. Those found in the cytoplasmic domain potentially prevent the dephosphorylation of their equivalent response regulators SilR and CusR leading to constitutive activation [23]. For strains carrying the *sil* operon, this is sufficient to achieve high level tolerance to silver containing compounds. However, the development of high-level silver tolerance through overexpression of the chromosomally located *cusCFBA* [12] operon requires additional, subsequent downregulation of the OmpC porin. This appears to be irrespective of whether the other dominant *Klebsiella* porin OmpF is already disrupted, e.g. in strain CFI_124_NDM1OXA232 (1 bp insertion in *ompF*). This suggests that OmpF does not play a significant role in silver ion uptake into cells whilst OmpC mutations presumably restrict the access of silver through the outer membrane and can mediate high level tolerance in conjunction with the CusS mutations. Mutations in OmpC alone are not sufficient for high level silver tolerance and *K. pneumoniae* organisms with mutations in one or both major porins alone do not appear to be intrinsically more silver tolerant than those with intact porins. Indeed, strains with mutations in SilS do not appear to alter their *ompC* expression levels suggesting that only *silCBA* overexpression is required for high level silver tolerance. This data suggests that SilCBA is a more efficient mechanism for reducing the intracellular silver ion concentration. This could be because the rate of efflux through CusCBA is reduced relative to SilCBA, either due to an increased copy number of the plasmid based SilCBA or that SilCBA has an increased affinity for Ag^+^ relative to CusCBA and is thus a higher affinity silver transporter. There is a precedence to mutate SilS where this option is available but the presence of the *sil* operon alone does not appear to result in strains with high intrinsic silver tolerance relative to strains lacking this operon. The clear advantage of *sil* operon carriage was when *K. pneumoniae* was challenged with a supra-MIC dose of AgNO_3_, presumably because it is far more probable to survive after one mutational event rather than having to mutate two genes. The indication that the sensor kinase (CusS) is mutated before the porin (OmpC) was interesting and again suggests that CusCBA is a lower affinity silver transporter and that increased efflux through this pump will act as an intermediate for development of higher tolerance. This is often the case with other antimicrobials such as fluoroquinolone resistance in *S. aureus* [24, 25] or increased chlorhexidine tolerance in *K. pneumoniae* [26], where mutations in efflux pump regulators precede the emergence of mutations in other target-related genes.

CusCBA and SilCBA are RND type efflux pumps but the addition of the efflux pump inhibitor PaβN did not have an effect on the tolerance to silver in either the wild-type strains or the silver-adapted strains indicated that it does not appear to interact with these efflux pumps. The addition of CCCP did reduce the silver tolerance for the pre-adapted strains but not for most adapted strains, irrespective of the causative silver tolerant mutation(s). We did attempt to increase the concentration of AgNO_3_ to see if this was simply due to higher tolerance but still saw no effect at the highest concentration tested (4096 mg/L). Above this concentration the solubility of AgNO_3_ becomes an issue. However, CCCP is a proton motive force disrupter and therefore has the potential to prevent influx of silver ions as well as blocking efflux. It also can make cells metabolically inactive and thus the synergy between silver and CCCP may be due to decreased metabolic activity rather than decreased efflux alone. Why two of the adapted strains (KPTR8 Ag and CFI_014_VIM4 Ag) were affected is unknown and does not appear to be reflected in the observed mutations. The effects of EPI’s upon silver tolerance are perhaps a little difficult to measure because one of the modes of action of silver ions is through disrupting membrane integrity [27] which may explain why the addition of the membrane permeabilizer PMBN also had no effect on the MIC’s to silver compounds. It might help to explain why strains with disrupted porins do not appear to be any less susceptible to silver than those with intact porins, although there is clearly a role for OmpC in silver uptake. OmpC plays an important role in the maintenance of membrane integrity [28] particularly when the bacterial membrane is placed under stress [29] and has a role in the transport of specific antibiotics [30]. It would be interesting to understand whether strains with pre-existing OmpC mutations that do not possess plasmids with the *sil* operon are better able to survive exposure to lethal silver concentrations. Unfortunately, we do not possess such a strain in our collection.

Silver coated dressings are often used in wound care such as burns [31, 32] and can be successfully used to treat MDR infections [33]. Our study shows that those strains which had increased silver tolerance were unaffected by any of the silver containing products tested corroborating previous studies [34, 35]. Silver nanoparticles have been shown to possess activity against Gram-negative bacteria in biofilms such as those that might be found in infected wounds [36, 37]. In this study, none of the silver containing dressing showed any effect on the growth and proliferation of the increased silver tolerance *K. pneumoniae* strains, regardless of the concentration and composition of silver within the proprietary antimicrobial dressing. Silver tolerant bacteria may be readily isolated following treatment with silver [15, 38] suggesting the clinical relevance of these bacteria and that this could become problematic with the increased use of silver ions in healthcare settings.

## Conclusions

Our findings evidence that endogenous silver tolerance can be readily induced in *K. pneumoniae* regardless of *sil* operon carriage and that this level of susceptibility could significantly compromise silver-based treatment in the clinic. With increasing numbers of MDR infections, the emergence of wide-spread increased silver resistance could have severe implications for the use of infection prevention and control procedures and the long-term treatment of such infections.

## Methods

### Bacterial strains and culture conditions

All relevant characteristics of all *K. pneumoniae* strains used in this study are listed in Table 2 and Supplementary Table S1. All strains were grown in mueller hinton broth 2 (MH2) with aeration or on tryptic soy agar (TSA) at 37 °C unless otherwise stated. Detection of genes of interest was carried out by analysis of whole-genome sequences.

**Table 2:** Characteristics of *K. pneumoniae* ST14/15 strains with respect to presence of genes associated with increased silver tolerance

						Mode MIC/MBC (mg/L)			
Strain	ST	*sil* operon	*cus operon*	*ompC*^a^	*ompF*	Silver Nitrate (AgNO_3_)		Silver Sulfadiazine (AgSD)	
						MIC	MBC	MIC	MBC
NCTC 13443	14	Yes	Yes	GD insertion	Tn disrupted	16–32	64	32–64	128
KPUK04a	14	Yes	Yes	GD insertion		16–32	64	64–128	128–256
KPUK04b	14	Yes	Yes			16–32	64	32	128–256
KPTR8	14	Yes	Yes			32	32–64	64	128–256
CFI_001_VIM1	14	Yes (Poss IS1 Tn insertion in *silF*)	Yes	Tn in promoter		64	64	128	256
CFI_111_OXA232	14	No	Yes	GD insertion		32	32–64	256	256
CFI_112_NDM1	14	Yes	Yes	L290R		64–128	128	128–256	256
CFI_124_NDM1OXA232	14	No	Yes		1 bp insertion after n622	16	32–64	64	128–256
CFI_128_NDM1	14	No	Yes	GD insertion		32	32–64	256	256
CFI_133_OXA484	14	No	Yes	GD insertion	W230STOP	32	32–64	256	128–256
CFI_146_OXA48	14	Yes	Yes			64	64	128	128–256
KPUK01	15	Yes	Yes	Tn in promoter	Y242STOP	8	32	16	64
18	15	Yes	Yes			16	32–64	64	256
CFI_005_VIM4	15	No	Yes		Tn disrupted	16	32–64	128	128–256
CFI_006_VIM4	15	No	Yes		Tn disrupted	16	32	128	256
CFI_014_VIM4	15	Yes	Yes		Tn disrupted	16	64	32	128–256
CFI_086_VIM2	15	No	Yes		Tn disrupted	32	64	128	128–256
CFI_104_VIM2	15	No	Yes		Tn disrupted	32	32–64	128	256
CFI_123_NDM1OXA232	15	Yes	Yes			>512	>512	>512	>512
CFI_132_OXA48	15	Yes	Yes		Tn disrupted	64	64	256	128–256
CFI_134_NDM1	15	No	Yes		Tn disrupted	16	32–64	32–64	64
CFI_139_KPC2	15	No	Yes			16	32–64	128	128–256
CFI_145_NDM1	15	Yes (Poss IS5 Tn insertion in *silB*)	Yes			8–16	16–64	32	32–64

^a^GD insertion occurs after amino acid 135 and is thought to restrict the pore size for OmpC

### Determination of the minimum inhibitory concentration (MIC) and minimum bactericidal concentration (MBC)

MIC assays were carried out by a broth microdilution method using a 96-well plate (Sigma-Aldrich Ltd., Dorset UK) according to European Committee on Antimicrobial Susceptibility Testing (EUCAST) guidelines as previously described [39]. The OD_600_ was measured after 16–20 h using a FLUOstar Omega plate reader (BMG Labtech, Aylesbury, UK). MBC determination was measured by plating out onto TSA plates 10 µls of MIC dilutions from and including the MIC level and the subsequent three further higher biocide concentrations (where applicable). The efflux pump inhibitors Carbonyl Cyanide 3-Chlorophenylhydrazone (CCCP) and phenyl-arginine-b-naphthylamide (PaβN) with 1 mg/L MgSO_4_ final concentration were added at concentrations of 10 mg/L and 25 mg/L respectively. Polymyxin B Nonapeptide (PMBN) was added at a final concentration of 30 mg/L.

### Generation of decreased silver susceptibility using a stepwise method

Seven ST14/15 *K. pneumoniae* strains were selected for adaptation to AgNO_3_ and consisted of a mixture of isolates with and without the *sil* operon. All cultures were grown in MH2 overnight where they were diluted down to a starting OD_600_ of 0.01 in MH2 supplemented with AgNO_3_ at a concentration of quarter the Modal MIC value (4 mg/L) and incubated shaking at 37 °C and at 250 RPM. Every two days 30 µls of culture was subsequently transferred to 3 mls fresh MH2 containing double the previous AgNO_3_ concentration, up to a final experimental concentration of 128 µg/ml (8 × MIC). Surviving cultures were then plated out on TSA and then passaged on TSA 10 times in the absence of AgNO_3_ before being tested. To control against changes in AgNO3 tolerance potentially caused by repeated passaging all strains were passaged every two days in MH2 without AgNO_3_ alongside the adaptation experiment.

### Generation of decreased silver susceptibility by culturing in an above MIC dose

Cultures were set up as described in the previous section except that the starting culture contained AgNO_3_ at a concentration of 400 mg/L (>10 × the Modal MIC value). They were incubated at 37 °C with shaking at 250 RPM for two days. Surviving cultures were again plated out on TSA and passaged on TSA 10 times in the absence of AgNO_3_. For time kill experiments a starting OD_600_ of 0.01 in MH2 supplemented with AgNO_3_ at 64 mg/L was used. Viable counts were performed by plating on TSA plates at 1, 2, 4, 6 and 24 h post AgNO_3_ challenge.

### Real-time PCR

Overnight cultures in MH2 were backdiluted to an OD_600_ of 0.1 and grown until an OD_600_ of 0.5 was reached at 37 °C with shaking. Cultures were then harvested using RNA protect (Qiagen) and RNA was extracted using the RNAeasy minikit (Qiagen) according to manufacturer’s instructions. cDNA was synthesized and real-time PCR was carried out using a StepOnePlus real-time PCR system and Fast SYBR green master mix (Life Technologies) as previously described [26]. Data were analysed using StepOnePlus analysis software v2.3 (Applied Biosystems) using *gapA* as an internal control. Primers used were *cusA* (KPCusARTPCRF1 ATGGTGCCGATGACGCTAAT; KPCUsARTPCRR1 GATAATCAGCAGCGCTTCGC), *cusS* (KPCusSRTPCRF1 ATCAGCAACCTGCTGCTGTCGAA; KPCusSRTPCRR1 TCGCTCAACTGGATGGTCAC), *silA* (KPSilARTPCRF3 CCGCGTTGTTCATTAGCCTG; KPSilARTPCRR3 ACGTTTTCGTGAATGCCAGC), *silS* (KPSilSRTPCRF3 CACCTGCCCCTTCGTAATGT; KPSilSRTPCRR3 TCCAGTCGCGCATCAAGATT) *ompC* (KPOmpCRTPCRF3 CATCGCTCTACCGCTGAAAG; KPOmpCRTPCRR3 TCGCTGGAGCTTTCAGTGTT).

### Silver dressing susceptibility

The susceptibility of *K. pneumoniae* silver adapted strains to five silver-containing wound dressings (four clinically and one commercially available) was compared with a generic non-antimicrobial wound dressing. These dressings were available on the NHS supply chain and are listed in the British National Formulary (https://bnf.nice.org.uk). The dressings contained differing forms and concentrations of silver. Briefly, a culture at OD_600_ of 0.1 culture was streaked onto TSA plates using a sterile swab to give a lawn of confluent growth and 1 × 1 cm^2^ piece of each dressing was placed onto the plate. Plates were incubated at 37 °C for 20 h. Corrected zones of reduced growth (CZRG) were then determined by measuring the diameter of the zone of reduced growth around the area of the dressing. A mean CZRG was obtained from three distinct replicate experiments. An unpaired t-test was used to determine the significance of the difference between wildtype and adapted strains.

### Whole genome sequencing

This was carried out as previously described [26] and PHE Galaxy was used to analyse genetic changes [40]. MLST typing of strains was performed using the Institut Pasteur *K. pneumoniae* whole genome MLST database [41].

## Supplementary Information

**Addtional file 1.**

## Data Availability

All datasets are available from the corresponding author on reasonable request.

## Abbreviations

MDR: Multi-drug Resistant
MBC: Minimum Bactericidal Concentration
MIC: Minimum Inhibitory Concentration
MLST: Multi locus sequence typing
MH: Mueller Hinton
NCTC: National Collection of Type Cultures
ORF: Open Reading Frame
TSA: Typtic Soy Agar
XDR: Extensively-Drug Resistant
