# Supplementary material for: Mutations in SilS and CusS/OmpC represent different routes to achieve high level silver ion tolerance in Klebsiella pneumoniae
Source: BMC Microbiol. 2022 Apr 25;22:113. doi: 10.1186/s12866-022-02532-y (PMC9036812; doi:10.1186/s12866-022-02532-y)
Supplement: Supplementary file 1 — Addtional file 1. [file 12866_2022_2532_MOESM1_ESM.docx]

Supplementary Table S1 – Non ST14/15 *K. pneumoniae* strains used in this study

|  |  |  |  |  |  | Mode MIC (mg/L) | |
| --- | --- | --- | --- | --- | --- | --- | --- |
| Strain | Sequence type | *sil* operon | *cus* operon | *ompC* (*ompK36*) mutation | *ompF* (*ompK35*) mutation | AgNO_3_ | AgSD |
| 82782 | 23 | No | Yes |  |  | 16 | 32 |
| 83498 | 23 | Yes | Yes |  |  | 16 | 32 |
| M109 | 23 | Yes | Yes |  |  | 16 | 32 |
| M6 | 23 | Yes | Yes |  |  | 16 | 64 |
| NCTC 14052 | 23 | Yes | Yes |  |  | 16 | 32 |
| TW3 | 23 | Yes | Yes |  |  | 16 | 32 |
| 12 | 35 | Yes | Yes | Q170STOP |  | 8 | 32 |
| M295 | 35 | Yes | Yes |  |  | 16 | 32 |
| M296 | 36 | No | Yes |  |  | 16 | 32 |
| 5041 | 37 | Yes (Tn insertion in *silS*) | Yes |  |  | 32 | 64 |
| 81066 | 37 | No | Yes |  |  | 16 | 64 |
| MGH78578 | 38 | Yes (frameshift in *silS*) | Yes |  | Upstream 108bp hypothetical protein insertion | >512 | 512 |
| 17 | 45 | Yes | Yes | Q170L | 13bp deletion | 32 | 256 |
| 83472 | 45 | No | Yes |  |  | 16 | 128 |
| 16 | 101 | Yes | Yes | Upstream TN insertion in promotor region |  | 32 | 128 |
| CFI_110_OXA232 | 101 | Yes | Yes |  | 1bp deletion | 128 | 256 |
| CFI_118_NDM1 | 101 | Yes | Yes |  | 1bp deletion | 32 | 256 |
| CFI_119_OXA32 | 101 | No | Yes |  | 1bp deletion | 16 | 128 |
| CFI_147_OXA48 | 101 | No | Yes |  | 1bp deletion | 16 | 128 |
| CFI_151_OXA48 | 101 | Yes | Yes |  | 1bp deletion | 64 | 128 |
| 6918 | 147 | Yes | Yes |  | Tn disrupted | 32 | 128 |
| 55850 | 147 | No | Yes |  |  | 32 | 64 |
| 45071 | 258 | Yes | Yes |  | 1bp insertion | 16 | 32 |
| 46704 | 258 | Yes | Yes |  | 1bp insertion | 16 | 32 |
| 49856 | 258 | Yes | Yes |  | 1bp insertion | 16 | 32 |
| 51851 | 258 | Yes | Yes |  | 1bp insertion | 16 | 32 |
| CFI_131_KPC2 | 258 | Yes | Yes |  | 1bp insertion | 32 | 32 |
| CFI_140_KPC3 | 258 | Yes (N33Y in *silA*) | Yes |  | 1bp insertion | 64 | 128 |
| CFI_141_KPC3 | 258 | Yes (N33Y in *silA*) | Yes |  | 1bp insertion | 64 | 128 |
| CFI_147_KPC2 | 258 | Yes | Yes |  | 1bp insertion | 64 | 64 |
| MKP103 | 258 | Yes | Yes |  | 1bp insertion | 32 | 128 |
| NCTC 13438 | 258 | Yes | Yes |  | 1bp insertion | 32 | 64 |
| M3 | 336 | Yes | Yes |  |  | 16 | 32 |
| 15 | 353 | No | Yes |  |  | 16 | 128 |
| 84052 | 567 | Yes (poss del in *silS*) | Yes |  |  | 32 | 64 |
| 11 | 1688 | No | Yes |  |  | 32 | 256 |
| M176 | N-A | No | Yes |  |  | 8 | 32 |
| CFI_130_NDM1 | Unknown | No | Yes |  |  | 64 | 128 |

Supplementary Table S2

| Strain | Fold upregulation | | | | | Mutations |
| --- | --- | --- | --- | --- | --- | --- |
|  | *cusA* | *cusS* | *silA* | *silS* | *ompC* |  |
| 13443 Ag | 41.6 | 3.45 | 298.4 | 18.1 | 2.04 | SilS A13V |
| KPUK04a Ag | 1.1 | 2.54 | 387.8 | 18.8 | 1.07 | SilS L322Q |
| CFI_124_NDM1OXA232 Ag | 1571.5 | 12.1 | - | - | 0.028 | CusS L9Q, transposon insertion in OmpC |
| CFI_134_NDM1 Ag | 276.5 | 3.09 | - | - | 0.018 | CusS P273L, OmpC deletion |

**Supplementary Table S2 – Expression levels of select genes with respect to wild-type levels following AgNO_3_ adaptation.** Results show that *ompC* is downregulated 35.7-fold in CFI_124_NDM1OXA232 Ag and 55.5-fold in CFI_134_NDM1 Ag respectively.

Supplementary Table S3 – Amino acid changes in the two-component systems SilRS and CusRS in wild-type (unadapted) strains compared to the consensus sequence.

| Strain | ST | SilR | SilS | CusR | CusS |
| --- | --- | --- | --- | --- | --- |
| NCTC 13443 | 14 |  |  |  |  |
| KPUK04a | 14 |  |  |  |  |
| KPUK04b | 14 |  |  |  |  |
| KPTR8 | 14 |  |  |  |  |
| CFI_001_VIM1 | 14 | S68T |  |  | A319T |
| CFI_111_OXA232 | 14 | Absent | Absent |  |  |
| CFI_112_NDM1 | 14 |  |  |  |  |
| CFI_124_NDM1OXA232 | 14 | Absent | Absent |  |  |
| CFI_128_NDM1 | 14 | Absent | Absent |  |  |
| CFI_133_OXA484 | 14 | Absent | Absent |  |  |
| CFI_146_OXA48 | 14 |  |  |  |  |
| KPUK01 | 15 |  |  |  |  |
| 18 | 15 |  |  |  |  |
| CFI_005_VIM4 | 15 | Absent | Absent |  |  |
| CFI_006_VIM4 | 15 | Absent | Absent |  |  |
| CFI_014_VIM4 | 15 |  |  |  |  |
| CFI_086_VIM2 | 15 | Absent | Absent |  |  |
| CFI_104_VIM2 | 15 | Absent | Absent |  |  |
| CFI_123_NDM1OXA232 | 15 | S68T, L128F, K129R, V135M, M220I, I223A | K41N, S59N, A66V, E70D, V73I, E355K |  |  |
| CFI_132_OXA48 | 15 |  |  |  |  |
| CFI_134_NDM1 | 15 | Absent | Absent |  |  |
| CFI_139_KPC2 | 15 | Absent | Absent |  |  |
| CFI_145_NDM1 | 15 | S68T, L128F, V135I, G207E, I223V | K41N, S59N, A66V, E70D, V73I, A102T |  |  |

Supplementary Table S4

|  | Mode MIC (mg/L) | | | | | | | | |
| --- | --- | --- | --- | --- | --- | --- | --- | --- | --- |
|  | AgNO_3_ | | | | | AgSD | | | |
| Chemical added | None | CCCP | PMBN | PAβN | None | | CCCP | PMBN | PAβN |
| NCTC 13443 | 32 | **2** | 32 | 32 | 128 | | **8** | 128 | 128 |
| NCTC 13443 Ag | >512 | >512 | >512 | >512 | >512 | | >512 | >512 | >512 |
| KPUK04a | 32 | **2-4** | 32 | 32-64 | 128 | | **16** | 64 | 128-256 |
| KPUK04a Ag | >512 | >512 | >512 | >512 | >512 | | >512 | >512 | >512 |
| KPTR8 | 64 | **2** | **16** | 32 | 128 | | **8** | 32 | 64-128 |
| KPTR8 Ag | >512 | **8** | >512 | >512 | >512 | | **32** | >512 | >512 |
| 18 | 32 | **8** | 16 | 32 | 64 | | **16** | 32 | 64 |
| 18 Ag | >512 | >512 | >512 | >512 | >512 | | >512 | >512 | >512 |
| CFI_014_VIM4 | 16 | **2** | 16 | 16-64 | 128 | | **8** | 32 | 64-128 |
| CFI_014_VIM4 Ag | >512 | **8** | >512 | **>**512 | >512 | | **16** | >512 | >512 |
| CFI_124_NMD1OXA232 | 8-32 | **4** | 16 | 16-32 | 64 | | **16** | 32-128 | 32-64 |
| CFI_124_NDM1OXA232 Ag | >512 | >512 | >512 | >512 | >512 | | >512 | >512 | >512 |
| CFI_134_NDM1 | 16 | **2** | 16 | 16 | 64 | | **8** | 64 | 32-64 |
| CFI_134_NDM1 Ag | >512 | >512 | >512 | >512 | >512 | | >512 | >512 | >512 |

**Supplementary Table S4 – Effect of EPI’s on tolerance to silver for *K. pneumoniae* strains which were adapted in a stepwise manner.** Ag after the strain name indicates that the strain was adapted to AgNO_3_. Values in bold indicate >2 log change when compared to strains exposed to AgNO_3_ and AgSD only. All MIC’s were performed in triplicate with the mode shown.

Supplementary Figure S1

**Supplementary Figure S1 – Growth analysis of AgNO_3_ adapted strains (Ag) relative to pre silver challenge strains.** Strains were grown in MH2 media at 37°C and OD_600_ values were taken every hour for 20 h.

Supplementary Table S5

| MIC's for Silver adapted and pre-exposure strains | GEN | TOB | AMK | CTZ | MEM | CIP | CST | PMB | TGC | DOX | CHL | RIF | OCT | CHD | ALX | DDAB |
| --- | --- | --- | --- | --- | --- | --- | --- | --- | --- | --- | --- | --- | --- | --- | --- | --- |
| NCTC 13443 | >512 | >512 | >512 | >64 | >64 | >64 | 0.5-1 | 1 | 8 | 8-16 | 512 | >512 | 2 | 16-32 | 1 | 4 |
| NCTC 13443 AgNO_3_ SW | >512 | >512 | >512 | >64 | >64 | >64 | 0.5 | 0.25-0.5 | 8 | 8-16 | 512 | >512 | 2 | 16-64 | 1 | 4 |
| NCTC 13443 AgNO_3_ HD | >512 | >512 | >512 | >64 | >64 | >64 | 0.5 | 0.5 | 8 | 8-16 | 512 | >512 | 2 | 16-32 | 1 | 4 |
| KPUK04a | 128 | 64 | 16 | >64 | 32 | >64 | 1 | 2 | 4 | 16 | 256 | >512 | 2 | 16-32 | 2 | 8 |
| KPUK04a AgNO_3_SW | 128 | 64 | 16 | >64 | 32 | >64 | 0.5-1 | 2 | 4-8 | 16 | 128-256 | >512 | 2 | 16-32 | 2 | 8 |
| KPUK04a AgNO_3_HD | 128 | 64 | 16 | >64 | 32 | >64 | 0.5-1 | 2 | 4 | 16 | 128-256 | >512 | 2-4 | 16-32 | 2 | 8 |
| KPTR8 | 512 | 64-128 | 4 | 1 | ≤0.06 | 0.25 | 0.5-1 | 1-2 | 16 | 128 | 128 | 32 | 4-8 | 16-32 | 2 | 32 |
| KPTR8 AgNO_3_SW | 512 | 256 | 4 | 0.5-1 | ≤0.06 | 0.125 | 0.5-1 | 1 | 16 | **16** | 64-128 | 32 | 8 | **4** | 2-4 | **8** |
| KPTR8 AgNO_3_HD | 256-512 | 128 | 4 | 1 | ≤0.06 | 0.25 | 0.5 | 1 | 16 | 128 | 64-128 | 32 | 8 | 8 | 2-4 | 32 |
| 18 | 1 | 1 | 2 | 1 | ≤0.06 | >64 | 1 | 0.5 | 2 | 32-64 | 128 | 16 | 4-8 | 16-32 | 2-4 | 16 |
| 18 AgNO_3_ SW | 1 | 2 | 4 | 1 | ≤0.06 | >64 | 0.5-1 | 0.5 | 4 | 32 | 128 | 16 | 4 | 16-32 | 4 | 16 |
| 18 AgNO_3_ HD | 1 | 1 | 4 | 1 | ≤0.06 | >64 | 1-2 | 2 | 4 | 32 | 128 | 16 | 4 | 16 | 4 | 16 |
| CFI_014_VIM4 | 256-512 | 32 | 8-16 | >64 | 32 | >64 | 0.5-2 | 0.5-2 | 0.5 | 16-32 | 8-16 | 8 | 2 | 16-32 | 2 | 4 |
| CFI_014_VIM4 AgNO_3_ SW | 512 | 32 | 16 | >64 | 32 | >64 | 1-4 | 1-4 | 0.5 | 32-64 | 16 | 8 | 4 | 16 | 1 | 4 |
| CFI_014_VIM4 AgNO_3_ HD | 512 | 64 | 8 | >64 | 32 | >64 | 1 | 2 | 0.5 | 64 | 8 | 8 | 4 | 16 | 1 | 4 |
| CFI_124_NDM1_OXA232 | 512 | >512 | >512 | >64 | 64 | >64 | 0.5 | 0.5 | 4 | 8-16 | 64 | 8 | 4 | 16-32 | 1 | 8 |
| CFI_124_NDM1_OXA232 AgNO_3_ SW | 512 | >512 | >512 | >64 | >64 | >64 | 0.25 | 1 | 4 | 8 | 64 | 4 | 2 | 8-16 | 1 | 8 |
| CFI_134_NDM1 | 512 | 512 | >512 | >64 | 32 | >64 | 0.25-0.5 | 0.25-0.5 | 4 | 8-16 | 512 | 8 | 4 | 4-8 | 1-2 | 16 |
| CFI_134_NDM1 AgNO_3_ SW | 512 | 512 | >512 | >64 | 64 | >64 | 0.25-0.5 | 1 | 4 | 16 | 512 | 4 | 2 | 8 | 1 | 16 |

**Supplementary Table S5 – MIC values of select antimicrobials against AgNO_3_ adapted strains compared to un-adapted strains.** GEN; gentamicin, TOB; tobramycin, AMK; amikacin, CTZ; ceftazidine, MEM; meropenem, CIP; ciprofloxacin, CST, colistin; PMB; polymyxin B, TGC, tigecycline; DOX, doxycycline, CHL, chloramphenicol; RIF; rifampicin, OCT; octenidine, CHD, chlorhexidine digluconate; ALX; Alexidine, DDAB; didodecyldimethylammonium bromide. Values in bold indicate >2 log change when compared to the non-silver exposed strain. All values shown are the mode of at least three repeats and are measured in mg/L.
